# Hidden ileostomy as a rescue procedure in major colorectal surgeries: a novel technique to prevent re-laparotomy in anastomotic leak cases

**DOI:** 10.1186/s40792-021-01259-8

**Published:** 2021-10-21

**Authors:** Mohsen Ezzy, Moustafa Elshafei, Khalid Hamdi, Thomas Kraus, Peter Heinz, Michael Svoboda, Katrin Fleischer, Kersten Grimm, Stefan Berkhoff

**Affiliations:** Department of General and Minimal Invasive Surgery, Nordwest Hospital, 60488 Frankfurt, Germany

**Keywords:** Hidden ileostomy, Colorectal anastomotic leak, Colorectal surgery, Complicated sigmoid diverticulitis, Rectal cancer, Sigmo-vesical fistula, Protective ileostomy

## Abstract

**Background:**

Anastomotic leak is a major cause of morbidity and mortality of patients worldwide, and it has remained stable over the last years. Routine construction of protective ileostomy is associated with stoma and negatively affects patients’ quality of life. Developing another technique to minimize those drawbacks with at least the same clinical success can help patients with anastomotic leak. We present the novel technique “Hidden Ileostomy” as an alternative to protective ileostomy that can achieve that balance.

**Materials and methods:**

Eight patients presented to our department underwent the novel technique “Hidden Ileostomy” as a rescue procedure for different reasons. The associated risk factors and clinical scenarios, together with the follow-up data, are presented.

**Results:**

For the eight cases in this study, one patient was ASA grade 1, 3 patients were classified as ASA grade 2, and 4 were grade 3. The mean ± SD operative time and blood loss were 196.3 ± 16.4 min and 325 ± 204.6 ml, respectively. The hidden ileostomy was removed after an average of 8 days. Only Case 6 reported an anastomotic leak on a postoperative day 10.

**Conclusion:**

A hidden ileostomy is an alternative and feasible technique in selected cases in colorectal surgery. This technique could be adopted in our practice instead of routine instruction of ileostomy, especially in the equivocal anastomosis.

## Introduction

Even after years of advances in surgery, anastomotic complications remain a challenge after colon and rectal resections. Anastomotic leak (AL) prevalence has been reported to be between 8.1 and 11% after colon and rectal resections [1, 2] with a mortality rate of 18.6% [3].

The mean length of hospital stay in patients with AL was 31.0 ± 47.1 days and median was 20 days, in comparison to those patients who did not have a postoperative leak was 18.3 ± 25.4 days and median was 13 days [4].

Despite the human and financial costs caused by AL [3] and the efforts put in reducing its occurrence, the incidence of AL has not evolved in the last years. Further, preoperative prediction of AL and identification of at-risk patients are not accurate enough [5].

Microbiome is a new and up-and-coming field of research; recent evidence supports the hypothesis that AL might result from a local infective complication, resulting in impaired healing at the anastomotic level due to a local increase collagenase activity [6, 7].

More recently, fluorescence perfusion angiography has shown a widespread clinical use in assessing the blood supply to the anastomosis. Jafari et al. [8] reported that fluorescence perfusion angiography reduced AL from 18 to 6% after robotic-assisted anterior resection.

Several meta-analyses have confirmed the role of protective ileostomy concluded that the diverting stoma significantly reduces the morbidity and mortality rate associated with AL and the rate of reoperation after anterior resection for low rectal cancer [9].

However, fecal diversions have been associated with poor quality of life, stoma-related complications, and perioperative risk of stoma closure later on (10).

To optimize the therapeutic care of patients with colorectal anastomosis with the hope to minimize the morbidity associated with AL and to reduce the routine creation of lateral ileostomy we will construct a novel technique known as hidden ileostomy in cases of left-sided colectomy and high anterior resection where the risk of postoperative leak is equivocal such as in emergency surgery, patients with medium risk factors and multiple co-morbidities as well as patients who refuse fecal diversion.

## Patients and methods

Here we describe a case series of 8 cases in which the Hidden Ileostomy technique was used. All baseline characteristics and follow-up data are described. Table 1 summarizes the baseline characteristics of the patients, patients’ history, investigations, and previous therapeutic options.

**Table 1:** Shows baseline characteristics of the included participants

Cases	Age	Gender	Indication	ASA	BMI	Preoperative colonoscopy findings	Performed operation	Operative time	Type of anastomosis	Level of anastomosis
Case 1	45	Male	Perforated sigmoid diverticulitis with 4-quadrant purulent peritonitis	1	23	Non	Open segmental sigmoid colectomy	195 min	End to end, 31 mm double-stapling technique	At sacral promontory
Case 2	55	Male	Sigmo-vesical fistula	2	25	Fistula by 30 cm above the anorectal junction	Laparoscopic segmental sigmoid colectomy + defect repair at the urinary bladder	200 min	End to end, 31 mm double-stapling technique	Just below the sacral promontory
Case 3	84	Male	Sigmoid cancer	3	19	Sigmoid cancer by 35 cm above the anorectal junction	Laparoscopic oncologic high anterior resection	210 min	End to end, 31 mm double-stapling technique	Just below the sacral promontory
Case 4	81	Female	Hartmann reversal, post-open sigmoidectomy by perforation	3	27	Rectum stump by 14 cm	Open Hartmann reversal	160 min	End to end, 25 mm double-stapling technique	Just below the sacral promontory
Case 5	70	Female	Chronic recurrent sigmoid diverticulitis	2	28	Sigmoid diverticulosis	Laparoscopic segmental high anterior rectum resection	192 min	End to end, 31 mm double-stapling technique	Just below sacral promontory
Case 6	82	Female	Sigmoid cancer	3	31	Rectosigmoid cancer 15 cm above the anorectal junction	Open oncologic high anterior resection	204 min	End to end, 31 mm double-stapling technique	Just below sacral promontory
Case 7	80	Female	Hartmann reversal, post-debulking op by peritoneal carcinomatosis by ovarian cancer	3	18	Rectum stump by 14 cm	Open Hartmann reversal, ileum segment resection	213 min	End to end, 25 mm double-stapling technique	Just below sacral promontory
Case 8	59	Male	Sigmoid stenosis by peritoneal metastasis of gastric cancer	2	26	Sub-obstructing sigmoid metastasis 35 cm ab anal verge	Open debulking surgery, oncologic sigmoid colectomy, 4 cm segmental resection of the ileum		Side to end handsewn anastomosis	At sacral promontory

Cases	Estimated blood loss (ml)	Intraoperative air leak test	Removal of hidden ileostomy	Postoperative coloscopy	Pathology	Neoadjuvant therapy	Loop related complication	Anastomotic leak	Hospital stay
Case 1	200	No leak	On OPD 8	Wide	Benign	No	No	No	10
Case 2	200	No leak	On OPD 9	Wide	Benign	No	No	No	11
Case 3	100	No leak	On OPD 8	Wide	Cancer, pT3 pN0 (0/14) L0 VO R0 cM0	No	No	No	10
Case 4	500	No leak	On OPD 8	Wide	Benign	No	No	No	10
Case 5	100	No leak	On OPD 7	Wide	Benign	No	No	No	10
Case 6	**500**	**No leak**	**On OPD 10**	**Anastomosis by 13 cm**	**Cancer, pT2 pN1b (3/12) L1 VO R0 cM0**	**No**	**No**	**Yes, on a postoperative day, 10**	28
Case 7	300	No leak	On OPD 9	Not done	Limited peritoneal carcinomatosis	No	No	No	**16**
Case 8	700	No leak	On operative day 9	Not done	Peritoneal carcinomatosis by gastric cancer	Yes, for gastric cancer, gastrectomy on 11.2016	No	No	17

### Case 1 (perforated sigmoid diverticulitis with 4-quadrant purulent peritonitis)

A 45-year-old male patient, a known case of sigmoid diverticulosis, ASA Grade 1, presented to our emergency department with a 1-week history of lower abdominal pain. Initially, the patient ignored this pain, but an acute exacerbation of pain accompanied by nausea and vomiting is brought to the emergency department. He denied any history of dysuria or alternation of bowel habits. On clinical examination, the patient was in pain with severe tenderness in the lower abdomen. The initial laboratory results show leukocytosis 15/NL, CRP 130 mg/l, and Natrium 135 mmol/l; other parameters were normal.

The emergency abdominal CT with IV contrast shows free air under the diaphragm and free fluid in the pelvis with fat stranding around the sigmoid colon, indicating free perforated sigmoid diverticulitis.

Informed consent was obtained, and the patient refused any kind of proximal fecal diversion. The patient underwent exploratory laparotomy; the diagnosis was confirmed with purulent peritonitis (Hinchey classification 3). Sigmoid segmental resection was performed with descendorectostomy at the sacral promontory level, and hidden ileostomy was instructed in the right lower abdomen. The operation took 195 min with 200 ml blood loss. Intraoperative air leak of bowel anastomosis shows no leak.

Postoperative sonography of the abdomen shows no complication related to hidden ileostomy such as ileus. Oral intake was gradually advanced as tolerated. The bowel motion was established. The silicon loop was removed on the eighth postoperative day. The patient was discharged on the postoperative 10th day in good general condition. The follow-up colonoscopy after 3 months shows intact, wide, and well-perfused anastomosis.

### Case 2 (sigmovesical fistula)

A 55-year-old male patient, known case of arterial hypertension, nicotine and alcohol abuse, chronic recurrent sigmoid diverticulitis, ASA Grade 2, referred to the outpatient clinic with recurrent UTIs pneumaturia, initially treated with multiple antibiotics therapy without improvement. He complained of mild colicky lower abdominal pain. He denied fever or shivering on physical examination, mild tenderness in the left lower abdomen and hypogastrium. The abdomen pelvis's MRI shows inflammatory reaction and wall thickness of the sigmoid colon and urinary bladder. The colonoscopy reveals sigmoid diverticulosis with mild mucosal inflammatory changes, fistula could be visualized by 30 cm above the anorectal junction. The laboratory results show leukocytosis 19/ NL, CRP 63 mg/l, and Natrium 135 mmol/l, nitric oxide urinary tract infection. The renal parameters were normal.

The diagnostic laparoscopy and sigmoid colectomy are indicated, and Informed consent was obtained. The proximal fecal diversion was not desired from the patient. The patient underwent diagnostic laparoscopy, intraoperative shows inflammatory adhesions and local inflammatory mass and fistula tract between the sigmoid colon and posterior wall of the urinary bladder, adhesiolysis, primary defect closure of urinary bladder and segmental sigmoid colectomy was done with primary anastomosis at level of sacral promontory, and hidden ileostomy was instructed in the right lower abdomen. The operation took 200 min with 200 ml blood loss. Intraoperative methylene blue test of the urinary bladder and air leak of bowel anastomosis shows no leak. Postoperative sonography of the abdomen shows no complication related to hidden ileostomy such as ileus. Oral intake was gradually advanced as tolerated. The bowel motion was established. The silicon loop is removed on the ninth postoperative day. The patient was discharged on the postoperative 11th day in good general condition. The follow-up coloscopy after 3 months shows intact, wide, and well-perfused anastomosis.

### Case 3 (adenocarcinoma of sigmoid colon)

An 84-year-old male patient, known case of arterial hypertension, insulin-dependent diabetes mellitus, coronary artery disease, atrial fibrillation, and hairy cell leukemia (first diagnosis 05/2010) in remission, ASA Grade 3, referred to the outpatient clinic with recently diagnosed sigmoid adenocarcinoma G2. He reports loss of appetite and weight loss from 8 kg over the last 6 weeks, body mass index (BMI) 19 kg/m^2^, lately complains of colicky lower abdominal pain, bleeding per rectum. He denies nausea or vomiting. On physical examination, he was cachexic; digital rectal examination reveals blood and mucous at the tip of the glove, no palpable masses. Preoperative staging shows circular wall thickness and mass in the sigmoid colon, with no distant metastasis. The colposcopy reveals fungating mass at 35 cm from the anal verge with partial luminal obstruction; the rest of the colon was normal. The laboratory results show hemoglobin of 10 g/dl, and tumor markers were not elevated, the liver and renal parameters were within the normal range.

The surgery was indicated, and informed consent was obtained. The proximal fecal diversion was not desired from the patient. The patient underwent laparoscopic high anterior rectum resection and partial TME with primary descendorectostomy just below sacral promontory, and hidden ileostomy was instructed in the right lower abdomen. The operation took 210 min with 100 ml blood loss. Intraoperative air leak test of anastomosis shows no leak.

The postoperative course was uneventful. Oral intake was gradually advanced as tolerated. The bowel motion was established. The silicon loop is removed on the eighth postoperative day. The patient was discharged on the postoperative 10th day in good general condition. The tumor stage was pT3 pN0 (0/14) L0 VO R0 cM0. The follow-up coloscopy after 3 months shows intact, wide, and well-perfused anastomosis.

### Case 4 (Hartmann reversal)

An 81-year-old female patient, ASA grade 3, a known case of type 1 diabetes mellitus and chronic renal insufficiency. The patient underwent the Hartmann procedure due to sigmoid perforation 6 months before. Her BMI was 27 kg/m^2^.

She admitted electively for Hartmann's reversal. On physical examination, she was in good general condition; the abdomen was soft and lax, without visible or palpable herniation. Digital rectal examination was unremarkable, and the rectal stump was not palpable. The colonoscopy revealed a rectum stump 14 cm from the anal verge; the rest of the colon was normal. The laboratory results show known chronic renal impairment.

We obtained informed consent from the patient after she had refused the fecal diversion option. She required reversal of Hartmann operation with an anastomosis just below the sacral promontory; during the reversal, a hidden ileostomy was performed at the right lower quadrant. The estimated operation time was 160 min, and the estimated blood loss was 500 ml. Intraoperative air leak of bowel anastomosis shows no leak. Postoperative sonography of the abdomen shows no complication related to hidden ileostomy such as ileus. Oral intake was gradually advanced as tolerated. The bowel motion was established. The silicon loop was removed on the eighth postoperative day. The patient was discharged on the postoperative 10^th^ day in good general condition. The follow-up colonoscopy after 6 weeks shows intact, wide, and well-perfused anastomosis.

### Case 5 (chronic recurrent sigmoid diverticulitis)

A 70-year-old female patient, ASA grade 2, a known case of arterial hypertension and obstructive sleep apnea, and chronic recurrent sigmoid diverticulitis (previous three attacks). The patient is admitted for elective laparoscopic sigmoid colectomy. BMI 28 kg/m^2^. The physical examination was otherwise unremarkable. The colonoscopy shows sigmoid diverticulosis; the rest of the colon was normal. The laboratory results show no pathology.

After the patient had reported disinterest in fecal diversion, we obtained informed consent to conduct our procedure. The patient underwent laparoscopic segmental high anterior rectum resection and anastomosis below sacral promontory, and a hidden ileostomy was performed at the right lower quadrant. The estimated operation time was 192 min, and the estimated blood loss was 100 ml. Intraoperative air leak of bowel anastomosis showed no leak. No complications (such as ileus) were noted via postoperative abdominal sonography. Oral intake was gradually advanced as tolerated. The bowel motion was established. The silicon loop was removed on the seventh postoperative day. The patient was discharged on the postoperative 10th day in good general condition. The follow-up coloscopy after 6 weeks shows intact, wide, and well-perfused anastomosis.

### Case 6 (sigmoid cancer)

An 82-year-old female patient, ASA grade 3, a known case of coronary artery disease, arterial hypertension, atrial fibrillation on rivaroxaban, arterial hypertension, chronic obstructive pulmonary disease, obstructive sleep apnea, hypothyroidism, and chronic renal insufficiency; BMI 31 kg/m^2^. She underwent four years ago to open sigmoidectomy, then Hartmann reversal secondary to sigmoid perforation. The patient has been recently diagnosed with colon cancer. On physical examination, the patient was in good general and performance status. No signs of bowel obstruction. The abdomen was soft and lax without visible or palpable herniation. The colonoscopy showed a rectosigmoid mass at 15 cm above the anal verge; the rest of the colon was normal. The laboratory results show Hb 10 g/dl and known chronic renal impairment.

CT thorax and abdomen revealed no distant metastasis. The patient refused fecal diversion; we obtained informed consent to conduct our procedure. The patient underwent open high anterior resection with primary anastomosis just below sacral promontory, and a hidden ileostomy was performed at the right lower quadrant. The estimated operation time was 204 min with 500 ml estimated blood loss. Intraoperative air leak of bowel anastomosis showed no leak. The initial postoperative course was uneventful. The patient reported on the 7th postoperative day low-grade fever, abdominal discomfort, and increased inflammatory marker. The abdominal sonography revealed no free fluid or ileus. We started the patient on intravenous antibiotic and hidden ileostomy kept in place. On 10th postoperative day, we noted feculent material in the abdominal drain as well as generalized abdominal tenderness. Emergency CT-abdomen with the intravenous and rectal contract was done, showed contrast extravasation at the anastomosis and free fluid collection in the pelvis. The patient underwent emergency sigmoidoscopy and proximal fecal diversion in operating theater. The endoscopic evaluation revealed anastomotic dehiscence less than 30% of anastomosis circumference. The hidden ileostomy is exteriorized through a 2–3 cm small incision. Both silicon ends are gently pulled out, and stoma is constructed in a standard fashion. The operative time was only 30 min. Postoperatively she continued on an intravenous antibiotic. The drain output was initially feculent with about 100 ml/24 h, then gradually become clear. Oral intake was gradually advanced as tolerated. The bowel motion was established. The patient was discharged on the postoperative 28th day in good general condition. The tumor stage was pT2 pN1b (3/12) L1 VO R0 cM0. The follow-up colonoscopy after 3 months shows healed and wide anastomosis.

### Case 7 (Hartmann reversal post-debulking secondary to ovarian cancer)

An 80-year-old female patient, ASA grade 3, a known case of arterial hypertension, type 2 diabetes mellitus, and coronary artery disease. The patient underwent debulking surgery with Hartmann procedure one year back by peritoneal carcinomatosis secondary to ovarian cancer. BMI 18 kg/m^2^, she admitted for elective Hartmann reversal upon her wish. On physical examination, the patient was emaciated. Colonoscopy revealed a rectum stump 14 cm from the anal verge; the rest of the colon was normal.

Imaging Studies showed limited peritoneal disease. The patient underwent the Hartmann reversal with primary anastomosis just below sacral promontory. Hidden ileostomy was constructed at the right lower quadrant. The estimated operation time was 213 min with 300 ml estimated blood loss. Intraoperative air leak of bowel anastomosis shows no leak. Oral intake was gradually advanced as tolerated. The bowel motion was established. The silicon loop was removed on the ninth postoperative day. The patient was discharged on the postoperative 16^th^ day in good general condition.

### Case 8 (sigmoid stenosis by peritoneal metastasis of gastric cancer)

A 59-year-old male patient, ASA grade 2, BMI 26 kg/m^2^. The patient has been diagnosed with gastric cancer on 10. 2016, he underwent neoadjuvant therapy with FLOT regimen then transhiatal gastrectomy. Postoperatively, he received adjuvant therapy. In 2020 he presented with abdominal pain and distension, no nausea or vomiting, or constipation. On physical examination, the abdomen was soft and lax but distended with high-pitched bowel sound. The CT-abdomen revealed bowel obstruction secondary to sigmoid stenosis. The patient was admitted, and a decompression gastric tube was placed. The next day we performed endoscopy and revealed a sub-obstructing sigmoid lesion 35 cm above anal verge; the rest of the colon was normal.

The biopsy was taken, the histology returned as metastasis of gastric cancer. The patient underwent a sigmoid colectomy with primary anastomosis at the sacral promontory level, and a hidden ileostomy was performed at the right lower quadrant. The estimated blood loss was 700 ml. Intraoperative air leak of bowel anastomosis showed no leak. No complications (such as ileus) were noted via postoperative abdominal sonography. Oral intake was gradually advanced as tolerated. The bowel motion was established. The silicon loop was removed on the ninth postoperative day. The patient was discharged on the postoperative 17th day in good general condition.

## Results and discussion

We present eight cases in this study, 4 (50%) were males, and 4 (50%) were females. The mean ± SD age of included patients was 69.5 ± 13.8 years, BMI was 24.65 ± 4.2 kg/m^2^. One patient was ASA grade 1, 3 patients were classified as ASA grade 2, and 4 were grade 3. The mean ± SD operative time and blood loss were 196.3 ± 16.4 min and 325 ± 204.6 ml, respectively. The hidden ileostomy was removed after an average of 8 days. Only Case 6 reported an anastomotic leak on a postoperative day 10. Table 1 shows a detailed illustration of the patients’ baseline characteristics, demographics, surgery indications, investigations, and operative related details.

**Fig. 1:**
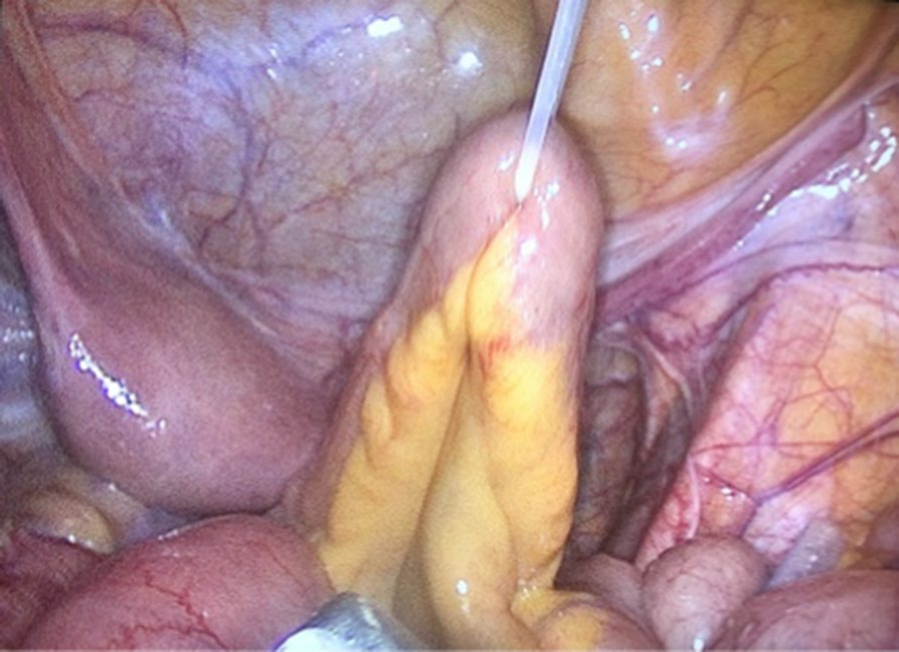
Hidden ileostomy: taping the ileum loop by passing a silicone tape

**Fig. 2:**
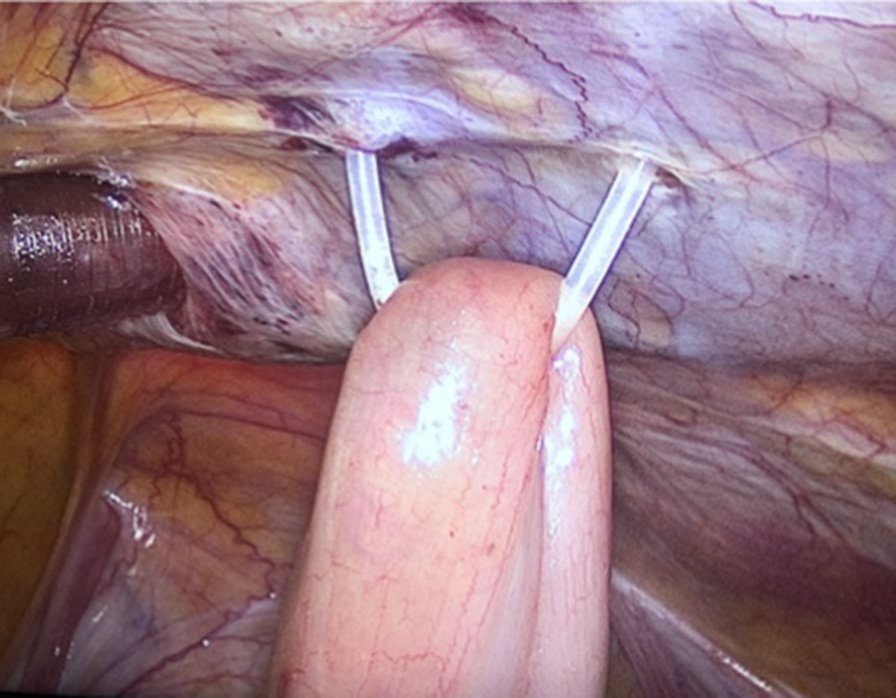
Hidden ileostomy: the hidden ileostomy is brought under the abdominal wall then the silicone loop is fixed to the skin in the right lower abdomen

**Fig. 3:**
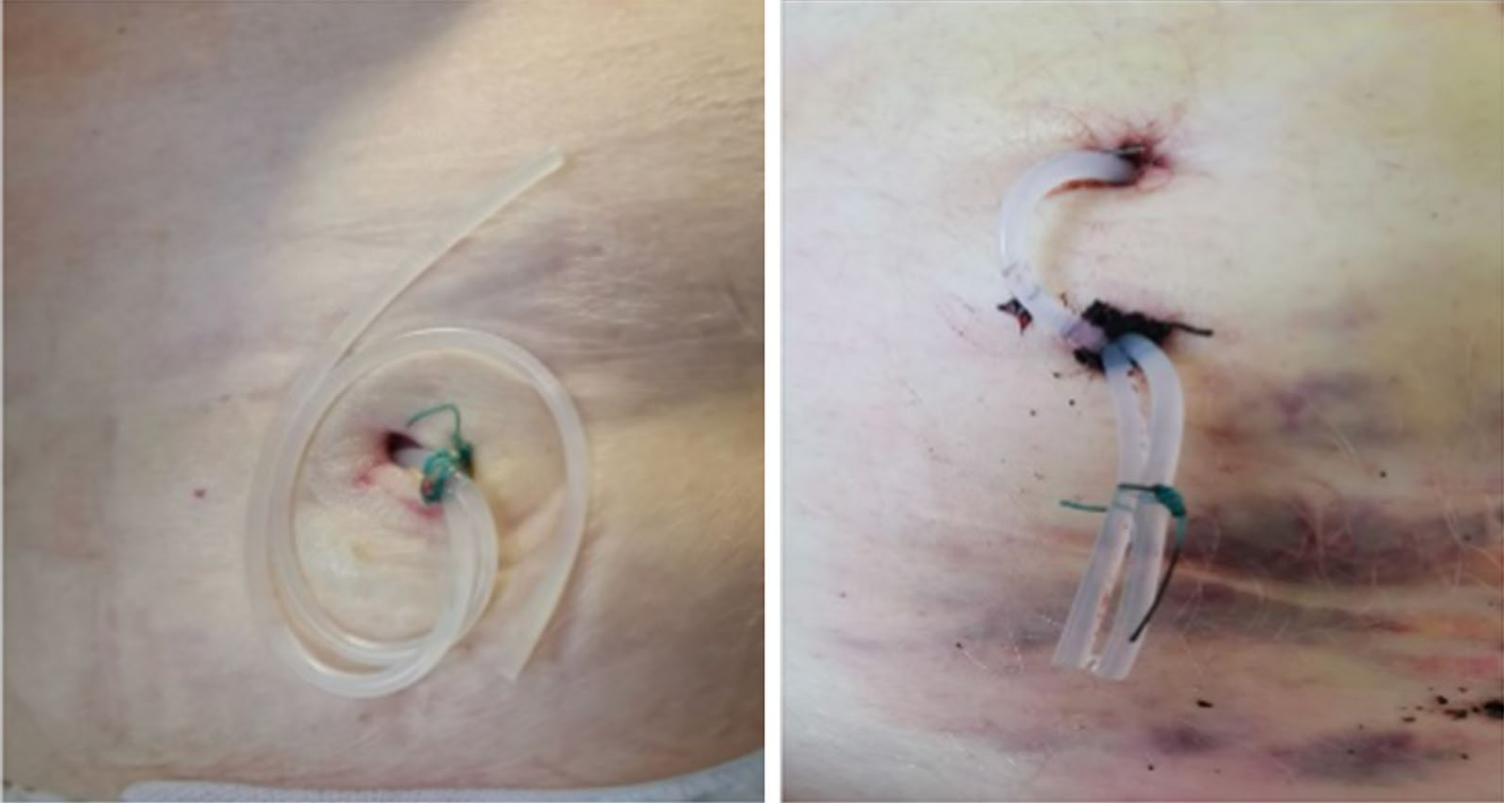
Hidden ileostomy: the silicone loop is fixed to the skin in the right lower abdomen

A hidden ileostomy is a stage before exteriorizing the ileostomy. After completing the major surgery, we locate the loop of the terminal ileum to construct the loop ileostomy. The loop selected for stoma should be at least 20 cm proximal to the ileocecal valve. For anatomical orientation, we marked the afferent loop with non-absorbable suture in the serosal layer. We taped the ileum loop by passing a silicone tape through a mesenteric window created close to the bowel, preferably in an avascular area as in Fig. 1. Both ends of silicone tape are exteriorized through a small incision through the abdominal wall (Fig. 2). Then we brought this loop ileostomy under the abdominal wall and left it unopened, making sure the loop is loose, not tight, then we fix both ends to the skin as in Fig. 3. This silicone loop will be covered with a sterile dressing.

If the patient develops a postoperative anastomotic leak, it is easy to exteriorize the pre-fashioned ileostomy through a 2–3 cm small incision. Both silicon ends will be gently pulled out, and the afferent loop is already marked with non-absorbable serosal suture for anatomical orientation, then stoma will be sutured to the skin in standard fashion. The intraoperative diagnostic endoscopy can be done to assess the extent of anastomotic leak and accordingly managed it. This procedure can be done under local anesthesia and sedation. So, the need for prolonged reoperation or unnecessary re-laparotomy could be avoided, and the risk of general anesthesia will be minimized without further morbidities to the patient.

If the patient’s postoperative course is uneventful and the patient has bowel motion and tolerated the oral intake, silicone tape can be removed on a postoperative day 5–10 and discharge the patient.

### Advantages

The technique could reduce the postoperative morbidity and mortality, and length of hospital stay of patients in cases of the leak. It may also help reduce the cost burden on the patient and avoid a redo laparotomy. This procedure can be done under local anesthesia and sedation without general anesthesia, which may further increase the patient’s morbidity and costs. This technique could spare a routine construction of loop ileostomy for many patients, avoid stoma-related complications, psychological impact, quality of life of the patients, and the risk associated with stoma closure.

### Disadvantages

If the silicon loop is too tight, it may cause postoperative small bowel obstruction, so intraoperatively we must be sure that the loop is loose and the bowel not under tension. Postoperatively patients must be monitored closely for any symptoms or signs of bowel obstruction. In suspected cases, the abdominal sonography could be an adjunct tool for further evaluation. The silicon loop is a foreign body-like drain, and it may be associated with the risk of Infection. So, local care and regular sterile dressing must be considered.

## Conclusion

A hidden ileostomy is an alternative and feasible technique in selected cases in colorectal surgery. This technique could be adopted in our practice instead of routine instruction of ileostomy, especially in the equivocal anastomosis.

## Data Availability

All data supporting this article are included in this manuscript.

## Abbreviations

ASA: American Society of Anesthesiologists
HI: Hidden ileostomy
SD: Standard deviation
AL: Anastomotic leak
CRP: C-reactive protein
UTIs: Urinary tract infections
BMI: Body mass index
